# Production of *ent*-kaurene from lignocellulosic hydrolysate in *Rhodosporidium toruloides*

**DOI:** 10.1186/s12934-020-1293-8

**Published:** 2020-02-05

**Authors:** Gina M. Geiselman, Xun Zhuang, James Kirby, Mary B. Tran-Gyamfi, Jan-Philip Prahl, Eric R. Sundstrom, Yuqian Gao, Nathalie Munoz Munoz, Carrie D. Nicora, Derek M. Clay, Gabriella Papa, Kristin E. Burnum-Johnson, Jon K. Magnuson, Deepti Tanjore, Jeffrey M. Skerker, John M. Gladden

**Affiliations:** 1 Department of Energy, Agile BioFoundry, Emeryville, CA 94608 USA; 2 Department of Biomass Science and Conversion Technology, Sandia National Laboratories, 7011 East Ave, Livermore, CA 94550 USA; 3 Advanced Biofuels and Bioproducts Process Development Unit, Lawrence Berkeley National Laboratory, Emeryville, CA 94608 USA; 4 Biological Systems and Engineering Division, Lawrence Berkeley National Laboratory, Berkeley, CA 94720 USA; 5 Earth and Biological Sciences Directorate, Pacific Northwest National Laboratory, Richland, WA 99354 USA; 6 Energy and Environment Directorate, Pacific Northwest National Laboratory, Richland, WA 99354 USA; 7 QB3-Berkeley, University of California, Berkeley, CA 94704 USA

**Keywords:** *Rhodotorula*, Mevalonate pathway, Diterpene, Geranylgeranyl pyrophosphate synthase, Mutant farnesyl pyrophosphate synthase, Metabolic engineering

## Abstract

**Background:**

*Rhodosporidium toruloides* has emerged as a promising host for the production of bioproducts from lignocellulose, in part due to its ability to grow on lignocellulosic feedstocks, tolerate growth inhibitors, and co-utilize sugars and lignin-derived monomers. *Ent*-kaurene derivatives have a diverse range of potential applications from therapeutics to novel resin-based materials.

**Results:**

The Design, Build, Test, and Learn (DBTL) approach was employed to engineer production of the non-native diterpene *ent*-kaurene in *R. toruloides*. Following expression of kaurene synthase (KS) in *R. toruloides* in the first DBTL cycle, a key limitation appeared to be the availability of the diterpene precursor, geranylgeranyl diphosphate (GGPP). Further DBTL cycles were carried out to select an optimal GGPP synthase and to balance its expression with KS, requiring two of the strongest promoters in *R. toruloides*, *ANT* (adenine nucleotide translocase) and *TEF1* (translational elongation factor 1) to drive expression of the KS from *Gibberella fujikuroi* and a mutant version of an FPP synthase from *Gallus gallus* that produces GGPP. Scale-up of cultivation in a 2 L bioreactor using a corn stover hydrolysate resulted in an *ent*-kaurene titer of 1.4 g/L.

**Conclusion:**

This study builds upon previous work demonstrating the potential of *R. toruloides* as a robust and versatile host for the production of both mono- and sesquiterpenes, and is the first demonstration of the production of a non-native diterpene in this organism.

## Background

The diverse terpenoid family contains over 70,000 unique compounds that are predominantly produced from two fundamental isoprenoid building blocks, the C5 prenyl phosphates, dimethylallyl diphosphate (DMAPP) and isopentenyl diphosphate (IPP), via the mevalonate (MVA) pathway or the 1-deoxyxylulose 5-phosphate (DXP) pathway [1–3]. These precursors are combined to generate longer prenyl phosphates such as the C10 monoterpene precursor, geranyl diphosphate (GPP), the C15 sesquiterpene precursor, farnesyl diphosphate (FPP), and C20 diterpene precursor, geranylgeranyl diphosphate (GGPP) [4, 5].

Diterpenes are less volatile than monoterpenes and sesquiterpenes, and have various industrial and biological applications from resin-based adhesives to potential new drugs [6–8]. In plants, diterpenes play diverse roles such as protection against pathogens and herbivores [9] and production of growth regulators such as gibberellins [10–12]. Plants, fungi and bacteria produce gibberellins from a universal precursor, *ent*-kaurene, which may be synthesized from GGPP in a single step or in two steps, via the intermediate *ent*-copalyl diphosphate (CDP) [13]. Gibberellins are used as a sustainable means of increasing yields and stress-tolerance in agriculture and floriculture but are currently expensive, leading to calls for biotechnological approaches to reduce cost and to increase the diversity of commercially available gibberellins [13, 14]. Besides serving as the precursor to gibberellins, *ent*-kaurene may also be transformed into a variety of bioactive derivatives, with a range of potential applications. Anti-microbial, anti-cancer, and anti-inflammatory activities are among the properties ascribed to kaurene-derived diterpenoids, such as *ent*-kaur-16-en-19-oic acid (kaurenoic acid) [15]. In traditional Chinese medicine, pharmacologically active *ent*-kaurene diterpenoids from *Isodon* plants (Lamiaceae) are used to treat inflammation and cancers [16]. Finally, kaurene-derivatives may play a key role in the development of new renewable polymers [17].

Only a few studies have reported on engineering microbes to produce *ent*-kaurene. For example, kaurene synthase (KS) from *Gibberella fujikuroi* was expressed in *Aspergillus nidulans* and although *ent*-kaurene production was demonstrated, it was not quantified [18]. In another study, CDP synthase and KS from *Stevia rebaudiana* were expressed in *Escherichia coli* along with three genes from the native DXP pathway, yielding 578 mg/L *ent*-kaurene from a glycerol-based (20 g/L) medium in a 1 L bioreactor [19]. To develop a truly sustainable solution for microbial production of bioproducts, the next step is to transition preliminary findings from studies like these to microbial hosts capable of utilizing cheap renewable carbon sources that do not compete with our food supply, such as lignocellulose.

Lignocellulose poses a challenge in that it is difficult to deconstruct into fermentable carbon, and many deconstruction technologies can produce toxic molecules that inhibit microbial growth and productivity. Much work has been done to develop technologies for efficient lignocellulose deconstruction and generation of non-toxic hydrolysates that are easily converted into bioproducts by microbial hosts capable of consuming lignocellulosic sugars. For example, a process was recently developed that generates clean lignocellulosic hydrolysates, called DMR-EH, with low concentrations of microbial growth inhibitors such as acetate, furfural, and 5-hydroxymethylfurfural and up to 230 g/L of monomeric sugars [20, 21]. This hydrolysate has been used to produce other terpene bioproducts, such as 1,8-cineole in a robust microbial host, *Rhodosporidium toruloides* [22].

*Rhodosporidium toruloides,* has emerged as a promising host for the utilization of lignocellulosic feedstocks, in part because it can withstand osmotic stress [23] and potential growth inhibitors found in biomass hydrolysates [24]. In addition, *R. toruloides* adapts well to the mixed carbon sources in low-cost lignocellulosic feedstocks, utilizing mixtures of C5 and C6 sugars in combination with lignin-derived compounds such as p-coumarate [25, 26]. *R. toruloides* can be grown to high cell densities, surpassing 150 g/L dry cell weight in high-gravity fermentation [27]. To date, *R. toruloides* has been engineered to produce a number of bioproducts, including lipids, the terpene biofuel candidates 1,8-cineole and bisabolene, and the non-ribosomal peptide, indigoidine [22, 26, 28, 29]. This work explores the potential for *R. toruloides* to produce diterpene products from lignocellulosic biomass, targeting the production of *ent*-kaurene from corn stover DMR-EH hydrolysate.

## Results

**Fig. 1:**
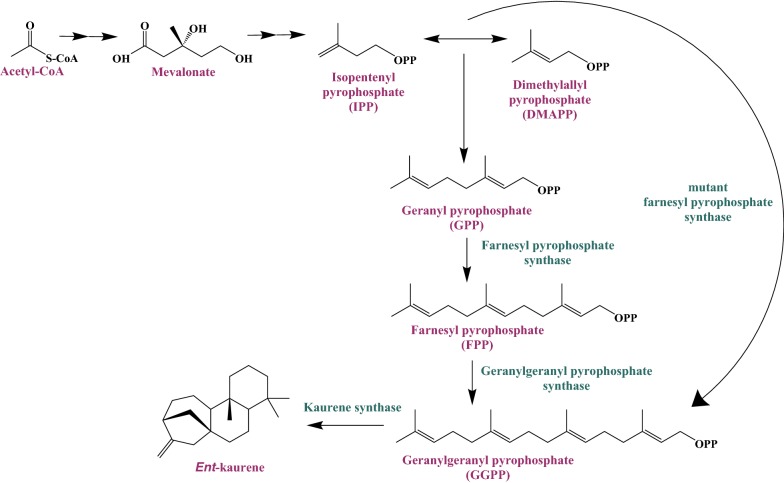
Production of *ent*-kaurene from acetyl-CoA via the mevalonate pathway. Genes expressed in *R. toruloides* are listed in Table 1. Mutant FPP synthases employed in this study are designed to alter prenyl phosphate product chain length, resulting in enzymes that generate mainly GGPP from IPP and DMAPP, instead of the native FPP product

To produce *ent*-kaurene in *R. toruloides*, we selected kaurene synthase from *Gibberella fujikuroi* (Gf*KS*) [30, 31] because it generates *ent*-kaurene directly from GGPP (Fig. 1) [13]. In plant and bacterial systems, *ent*-kaurene is synthesized from GGPP in a two-step process, via CDP, while *G. fujikuroi* and other fungi contain bifunctional CDP/KS enzymes that generate *ent*-kaurene directly from GGPP (Fig. 1) [13]. The native promoters *GAPDH* (glyceraldehyde 3-phosphate dehydrogenase) and *ANT* (adenine nucleotide translocase) were chosen for heterologous expression of GfKS based on analysis of RNAseq data from a previous study that suggests that they both are constitutive and drive a high level of gene expression [32].

**Fig. 2:**
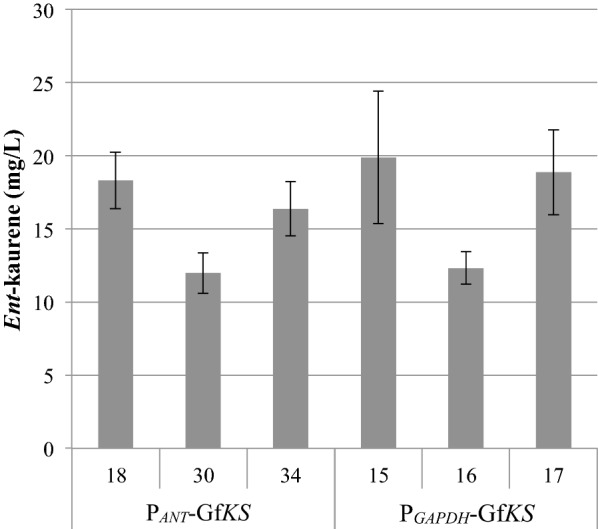
Expression of kaurene synthase from *Gibberella fujikuroi* (Gf*KS*) in *R. toruloides*. *Ent*-kaurene titer at 9 days is shown for the three highest-titer strains transformed with constructs 1 (P_*ANT*_-Gf*KS*) and 2 (P_*GAPDH*_-Gf*KS*). (N = 3, data shown as average ± standard deviation, from a single experiment in YPD_10_ medium. ABFPUB identification numbers are listed in Table 1.)

Constructs were transformed into *R. toruloides* by *Agrobacterium tumefaciens* mediated transformation (ATMT), which results in random chromosomal integration of transgenes. Typically, 40 transformants were selected for initial measurement of *ent*-kaurene production and then the three highest-titer strains for each construct were compared in triplicate. Following screening of strains transformed with constructs harboring P_*ANT*_-Gf*KS* (construct 1) and P_*GAPDH*_-Gf*KS* (construct 2), we found that similar maximum *ent*-kaurene titers of 15-20 mg/L were reached in YPD_10_ medium (YPD containing 100 g/L glucose; Fig. 2; constructs are listed in Table 1). The low *ent*-kaurene titers observed relative to other heterologous terpenes produced in this organism (typically several hundred mg/L) suggests that the availability of the Gf*KS* substrate, GGPP, may be limiting. Although *R. toruloides* produces carotenoids, they are produced at relatively low levels, indicating that native carbon flux to GGPP may be low [26].

**Table 1:** Overview of constructs used to engineer *ent*-kaurene production in *R. toruloides*

Plasmids	Genotypes/features	ABFPUB registry ID
Construct 1	P_*ANT*_-Gf*KS*-HYG^R^	ABFPUB_1
Construct 2	P_*GAPDH*_-Gf*KS*-NAT^R^	ABFPUB_3
Construct 3	P_*TEF1*_-Rt*ERG20*(F91C)-KanMX^R^	ABFPUB_6
Construct 4	P_*TEF1*_-Tc*GGPPS*(S239C,G29D)-KanMX^R^	ABFPUB_7
Construct 5	P_*TEF1*_-Gg*FPS*(F112A)-KanMX^R^	ABFPUB_8
Construct 6	P_*TEF1*_-Rt*BTS1*-KanMX^R^	ABFPUB_9
Construct 7	P_*ANT*_-Gf*KS*-P_*TEF1*_-Rt*ERG20*(F91C)-NAT^R^	ABFPUB_10
Construct 8	P_*ANT*_-Gf*KS*-P_*TEF1*_-Tc*GGPPS*(S239C, G295D)-NAT^R^	ABFPUB_11
Construct 9	P_*ANT*_-Gf*KS*-P_*TEF1*_-Gg*FPS*(F112A)-NAT^R^	ABFPUB_12
Construct 10	P_*ANT*_-Gf*KS*-P_*TEF1*_-Rt*BTS1*-NAT^R^	ABFPUB_13
**Strains**	IFO0880 (WT)	ABFPUB_14
	IFO0880/P_*GAPDH*_-Gf*KS*-NAT^R^	ABFPUB_15–17
	IFO0880/P_*ANT*_-Gf*KS*-HYG^R^	ABFPUB_18, 30, 34
	IFO0880/P_*GAPDH*_-Gf*KS*-NAT^R^/P_*TEF1*_-Rt*ERG20*(F91C)-KanMX^R^	ABFPUB_47–49
	IFO0880/P_*GAPDH*_-Gf*KS*-NAT^R^/P_*TEF1*_-Tc*GGPPS*(S239C,G29D)-KanMX^R^	ABFPUB_19, 20, 31
	IFO0880/P_*GAPDH*_-Gf*KS*-NAT^R^/P_*TEF1*_-Gg*FPS*(F112A)-KanMX^R^	ABFPUB_32, 33, 35
	IFO0880/P_*GAPDH*_-Gf*KS*-NAT^R^/P_*TEF1*_-Rt*BTS1*-KanMX^R^	ABFPUB_38–40
	IFO0880/P_*ANT*_-Gf*KS*-P_*TEF1*_-Rt*ERG20*(F91C)-NAT^R^	ABFPUB_41–43
	IFO0880/P_*ANT*_-Gf*KS*-P_*TEF1*_-Tc*GGPPS*(S239C,G295D)-NAT^R^	ABFPUB_21, 22, 29
	IFO0880/P_*ANT*_-Gf*KS*-P_*TEF1*_-Gg*FPS*(F112A)-NAT^R^	ABFPUB_23, 26, 36
	IFO0880/P_*ANT*_-Gf*KS*-P_*TEF1*_-Rt*BTS1*-NAT^R^	ABFPUB_44–46

Strains and plasmids used in this study are available upon request through the Agile BioFoundry Strain Registry (http://public-registry.agilebiofoundry.org// [37])

*GAPDH* glyceraldehyde 3-phosphate dehydrogenase, *ANT*, adenine nucleotide translocase, *TEF1* translational elongation factor, Gf*KS*, *Gibberella fujikuroi* kaurene synthase (NCBI Accession Number, Q9UVY5), *RtERG20(F91C)* mutant farnesyl pyrophosphate synthase from *R. toruloides* (PRQ75922.1), *TcGGPPS(S239C,G295D)* mutant geranylgeranyl pyrophosphate synthase from *Taxus canadensis* (AAD16018), Gg*FPS*(F112A) mutant farnesyl pyrophosphate synthase from *Gallus gallus* (P08836.2) and Rt*BTS1* geranylgeranyl pyrophosphate synthase *BTS1* from *R. toruloides* (PRQ72579)

**Fig. 3:**
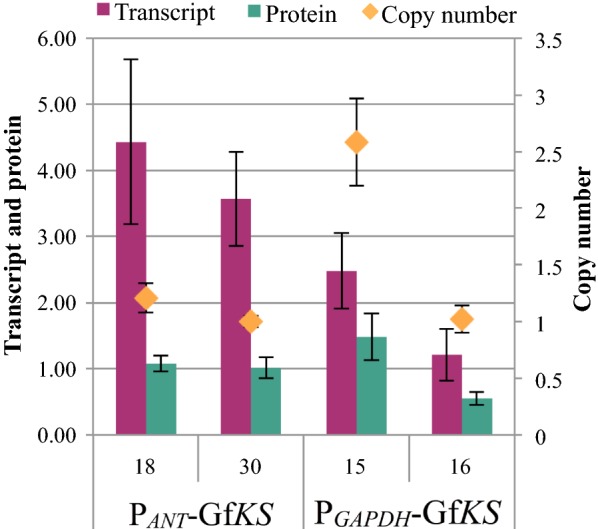
Transcript level, protein level and copy number of Gf*KS*. Average relative abundance of Gf*KS* transcript (ΔCt), protein, and copy number on day 3. Samples are designated by their ABFPUB identification number and description. (N = 3, data shown as average ± standard deviation, from a single experiment in YPD_10_ medium.)

Similar maximum titers were reached following transformation of *R. toruloides* with the P_*ANT*_-Gf*KS* and P_*GAPDH*_-Gf*KS* constructs, even though the *ANT* promoter is natively stronger, as indicated by *ANT* transcript levels and reporter studies [32]. To test whether this relative difference in promoter strength also applies to expression of the heterologous KS, Gf*KS* copy number, transcript levels, and protein levels were measured for selected Gf*KS* strains (Fig. 3). In strains harboring Gf*KS* at similar copy numbers, Gf*KS* transcript and protein levels were higher when expressed from P_*ANT*_ (strains ABFPUB_18 and 30) than from P_*GAPDH*_ (strain ABFPUB_16). In one P_*GAPDH*_-Gf*KS* strain, ABFPUB_15, Gf*KS* transcript and protein levels are higher, but this is likely due to the two- to threefold higher Gf*KS* copy number in ABFPUB_15.

Comparison of transcript and protein levels of KS with *ent*-kaurene titers can also give an indication of whether gene expression or metabolite pools are limiting *ent*-kaurene production. The P_*GAPDH*_-Gf*KS* strain ABFPUB_16 had the lowest transcript and protein levels of the strains examined. While other strains (e.g., the P_*ANT*_-GfKS strain ABFPUB_30) had two- to fourfold higher transcript and protein levels, no substantial improvement in *ent*-kaurene titers was observed (Figs. 2 and 3). This indicates that a further increase in KS expression is not likely to significantly improve *ent*-kaurene production.

**Fig. 4:**
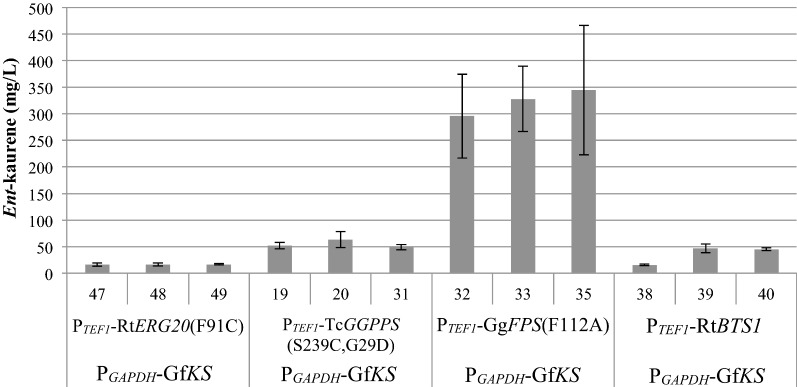
Co-expression of kaurene synthase from *Gibberella fujikuroi* (Gf*KS*) and various GGPP synthases in *R. toruloides*. Strains were constructed by transformation of strain ABFPUB_16 (P_*GAPDH*_-Gf*KS*) with constructs 3 to 6. *Ent*-kaurene titer at 9 days is shown for the three highest-titer strains for each construct. (N = 3, data shown as average ± standard deviation, from a single experiment in YPD_10_ medium. ABFPUB identification numbers are listed in Table 1.)

To investigate the possibility that GGPP levels may be a major factor limiting *ent*-kaurene titers, several GGPP synthases (*GGPPS*s) were selected for expression in a strain harboring Gf*KS*. ABFPUB_16 was selected as a base strain, as it ranked most consistently as a low-variance, top-titer strain among the 80 Gf*KS* transformants assayed in repeated screening trials. The native promoter *TEF1* (translational elongation factor 1) was chosen to express each of four candidate GGPPSs: the native *R. toruloides* GGPPS (construct 6, P_*TEF1*_-Rt*BTS1*); a mutant of the *R. toruloides* farnesyl pyrophosphate (FPP) synthase (construct 3, P_*TEF1*_-Rt*ERG20*(F91C)); a mutant GGPPS from *Taxus canadensis* (construct 4, P_*TEF1*_-Tc*GGPPS*(S239C, G295D)); and a mutant FPP synthase from *Gallus gallus* (construct 5, P_*TEF1*_-Gg*FPS*(F112A)). The mutations, F91C and F112A, introduced to the *R. toruloides* and *G. gallus* FPP synthases, respectively, are designed to alter prenyl phosphate product chain length, resulting in enzymes that predominantly generate GGPP instead of the native FPP product [33]. The mutations S239C and G295D were previously identified in a carotenoid-based screen for improvements to the *T. canadensis* GGPPS [34]. Overexpression of Rt*ERG20*(F91C) (construct 3) generally resulted in titers that were lower than or, at best, matching those of the parent strain ABFPUB_16, perhaps due to an unfavorable balance between FPP and GGPP biosynthesis, unintended enzyme inactivation due to mutation of the F91 residue, or feedback regulation of the native Rt*ERG20* gene in *R. toruloides* (Fig. 4). However, overexpression of either the native *R. toruloides* GGPPS (construct 6) or the mutant GGPPS from *T. canadensis* (construct 4) resulted in more than two- and threefold increases in *ent*-kaurene titer, respectively. The most successful strategy was overexpression of the mutant FPP synthase from *G. gallus* (construct 5), which yielded a 17-fold increase in *ent*-kaurene titer, to 345 mg/L, in YPD_10_ medium. These results indicate that overexpression of GGPPS increases the available pool of GGPP for KS, resulting in an improvement in *ent*-kaurene titers.

**Fig. 5:**
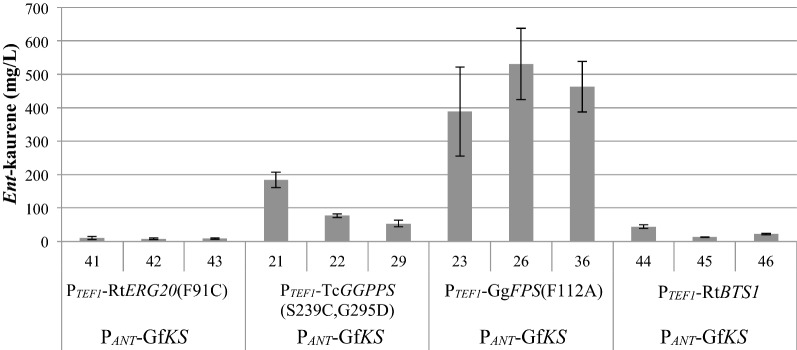
Co-transformation of kaurene synthase from *Gibberella fujikuroi* (Gf*KS*) and various GGPP synthases into WT *R. toruloides* (constructs 7–10, Table 1). *Ent*-kaurene titer at 9 days is shown for the three highest-titer strains for each construct. (N = 3, data shown as average ± standard deviation, from a single experiment in YPD_10_ medium. ABFPUB identification numbers are listed in Table 1.)

Optimizing carbon flux through a biosynthetic pathway requires balancing the expression of each pathway enzyme. The overexpression of GGPPS alongside KS shifted the balance of the pathway and resulted in an increase in *ent*-kaurene titers. To test whether this shift in pathway balance has caused KS to become a limiting factor, designs were made to balance KS and GGPPS expression by incorporating each P_*TEF1*_-GGPPS and P_*ANT*_-Gf*KS* in a 1:1 ratio in the same construct. P_*ANT*_ was chosen over P_*GAPDH*_ in an attempt to restore any potential deficit in KS expression in the stacked KS GGPPS strains. After transformation into WT *R. toruloides* by ATMT, relative performance of the four GGPPSs was observed to remain the same but higher absolute *ent*-kaurene titers were achieved with Gf*KS* expression under control of P_*ANT*_, suggesting that the KS may have become limiting as GGPP supply increased (Fig. 5). Co-transformation of P_*ANT*_-Gf*KS* with P_*TEF1*_-Tc*GGPPS*(S239C, G295D) (construct 8) and P_*TEF1*_-Gg*FPS*(F112A) (construct 9), resulted in *ent*-kaurene titers of 184 and 531 mg/L in YPD_10_ medium, respectively.

**Fig. 6:**
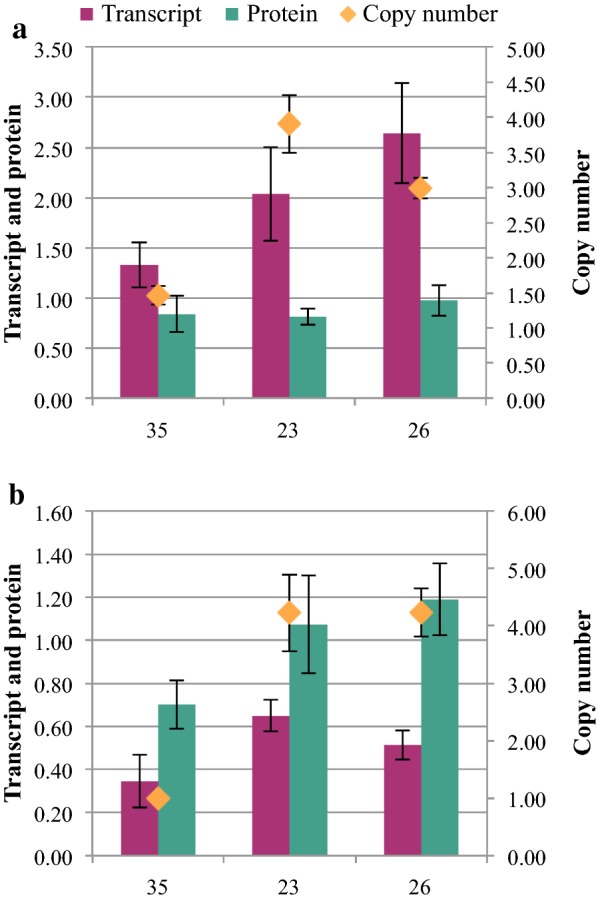
Average relative abundance of transcript (ΔCt), protein and copy number of **a** Gf*KS* and **b** Gf*FPS*(F112A) on day 3. Samples are designated by their ABFPUB identification number. (N = 3, data shown as average ± standard deviation, from a single experiment in YPD_10_ medium.)

In order to understand how co-expression of KS and GGPPS in a single construct affected expression of each of these enzymes relative to iteratively stacking individual constructs into *R. toruloides*, transgene copy number, transcript and protein abundances of Gf*KS* and Gg*FPS*(F112A) were compared in three high-titer strains (Fig. 6). Transgene copy number was three- to fourfold higher in strains ABFPUB_23 and ABFPUB_26, which were generated by transformation of a single construct, relative to strain ABFPUB_35, which was constructed by gene stacking—transformation of ABFPUB_16 with P_*TEF1*_-Gg*FPS*(F112A). Interestingly, Gf*KS* protein level is similar in all three strains, while and Gg*FPS*(F112A) protein level is higher in strains ABFPUB_23 and ABFPUB_26 than in strain ABFPUB_35. Overall, the highest average *ent*-kaurene titer was achieved in strain ABFPUB_26, which had the highest GgFPS(F112A) protein level.

**Fig. 7:**
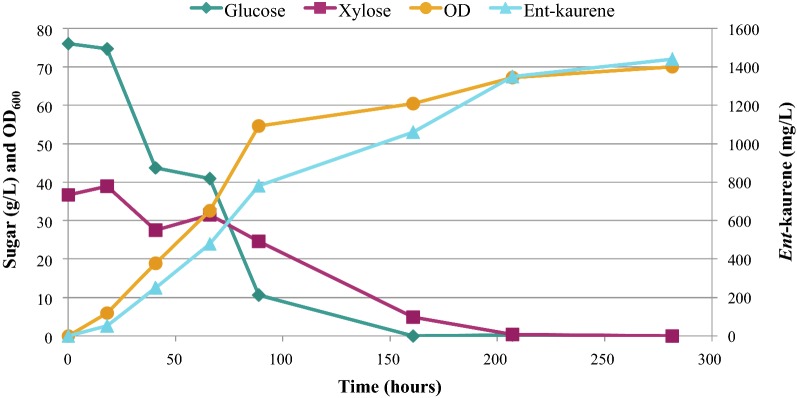
Sugar concentration, OD_600_, and *ent*-kaurene titer data for strain ABFPUB_26 cultivated in a 2 L bioreactor containing 75% DMR-EH hydrolysate, supplemented with 10 g/L yeast extract

One of the advantages of *R. toruloides* as a host is its ability to grow on lignocellulosic hydrolysates and to perform well when cultivated in bioreactors [26, 35]. To demonstrate production of *ent*-kaurene from lignocellulose, DMR-EH hydrolysate was prepared from corn stover as described previously [21]. Strain ABFPUB_26 (harboring P_*ANT*_-Gf*KS* and P_*TEF1*_-Gg*FPS*(F112A)) was selected for scale-up to a 2 L bioreactor in a medium composed of 75% corn stover DMR-EH hydrolysate, supplemented with 10 g/L yeast extract. Under these conditions, an *ent*-kaurene titer of 1.44 g/L was achieved (Fig. **7**). OD_600_ and titer increased proportionally, reaching an OD_600_ of 70 by 281 h; glucose was completely consumed by 161 h and xylose was fully consumed after 207 h. Strain ABFPUB_26 had produced *ent*-kaurene at 531 mg/L when cultivated in YPD_10_ medium in culture tubes, yet produced 2.7 fold more *ent*-kaurene when scaled up to a 2 L bioreactor and 75% DMR-EH growth medium, which contains only 13% more sugar (76 g/L glucose and 37 g/L xylose) than YPD_10_. This underscores the compatibility of *R. toruloides* with lignocellulosic feedstocks.

## Discussion

This work applies the Design, Build, Test, and Learn (DBTL) approach to engineer production of the non-native diterpene *ent*-kaurene in *R. toruloides*. The ATMT transformation method used in this study promotes random integration of constructs into the *R. toruloides* genome, generating strains that vary in transgene insertion site and copy number. The resulting variation in transgene expression level can be advantageous in that it adds an additional mechanism to adjust transgene expression in pathway engineering work. However, it also can confound direct comparison of different engineering strategies. Fortunately, this issue can be overcome simply by measuring transgene copy number. For example, a direct comparison between the ANT and GAPDH promoters was made in this study and we observed that P_*ANT*_ resulted in stronger *GfKS* expression than P_*GAPDH*_, in agreement with previous data comparing these promoters [32].

Interestingly, unlike previous studies that used these same strong promoters to express a sesquiterpene synthase to produce 100–500 mg/L of sesquiterpene, simply overexpressing *GfKS* resulted in almost tenfold lower titers of *ent*-kaurene [26]. Sesquiterpenes are produced from FPP (the immediate precursor of GGPP), which is a metabolite used for many cellular functions, including biosynthesis of essential sterols such as ergosterol. In contrast, GGPP is used primarily for producing low levels of non-essential carotenoids in *R. toruloides*. Therefore, it’s not surprising that this study found evidence that the apparent GGPP metabolite pool is lower than that of FPP. This was substantiated by the significant increase in *ent*-kaurene titers observed upon expression of a GGPPS. The variance in *ent*-kaurene titer in the GGPPS overexpression strains is relatively high, so it’s difficult to make conclusive comparisons, but the relative trend in titer suggests that higher levels of GgFPS(F112A) protein promoted higher *ent*-kaurene titers. These results suggest that the increased GGPPS expression level in the strains generated from a single construct in the final DBTL cycle created a better balance in the pathway, leading to higher flux toward *ent*-kaurene. Overall, the difference in titer in strains harboring GGPPS constructs was relatively modest, likely indicating that expression of these two terminal enzymes is reaching saturation.

While g/L titers of *ent*-kaurene were achieved by engineering two pathway steps, there are many improvements that can be made to further improve titer, rate and yield (TRY). Additional investigation of the impact of GGPPS expression on the balance between FPP and GGPP could prove fruitful, either from further promoter optimization for both KS and GGPPS expression or by examining other KS and GGPPS orthologs. Beyond these terminal steps in the biosynthetic pathway, optimization of the entire mevalonate pathway will likely lead to improvements in TRY. There are also several broader avenues to explore to facilitate engineering this nascent host. For example, little is known about the impact of integration locus on expression in *R. toruloides* and developing a better understanding of this topic is a high-priority for further investigation. Other engineering tools that would be highly beneficial for pathway optimization include methods to downregulate competing pathways, such as lipid biosynthesis. To that end, the application of RNAi has recently been demonstrated in *R. toruloides*, with downregulation of fatty acid synthases [36]. This tool will be valuable for future studies.

Outside of metabolic engineering approaches, process optimization of cultivation conditions will be important to improve heterologous terpene production in *R. toruloides*. For example, optimization of DMR-EH hydrolysate media to eliminate the use of the yeast extract in favor of a more industrially-relevant nitrogen source such as ammonium sulfate would be ideal. Also, identification of other vitamins and minerals that are limiting in DMR-EH hydrolysates will likely improve TRY. Finally, a deeper exploration of cultivation conditions such as pH, mixing, aeration, and temperature are also needed in this relatively new host organism. Even without these important optimizations, the final titer of 1.44 g/L achieved in this study is the highest reported *ent*-kaurene titer in any microbial cell factory.

## Conclusion

This study builds upon previous work demonstrating the potential of *R. toruloides* as a robust and versatile host for the production of both mono- and sesquiterpenes, and is the first demonstration of the production of a non-native diterpene in this organism. It provides a roadmap for rapid high-titer diterpene production in *R. toruloides*; it is possible that other diterpene synthases could be combined with the GGPPS *GgFPS*(F112A) in a single expression construct to achieve production of g/L quantities of the diterpene. These results, in combination with previous studies on mono- and sesquiterpenes, indicate that *R. toruloides* is an ideal host for the production of a range of different heterologous terpene bioproducts from cheap renewable carbon sources, such as lignocellulosic biomass.

## Materials and methods

### Plasmid design and construction

Plasmids were based on a binary vector for *Agrobacterium tumefaciens* mediated transformation (ATMT) containing a bacterial expression cassette conferring resistance to kanamycin and *R. toruloides* expression cassettes conferring resistance to nourseothricin (NAT), G418 (KanMX) or hygromycin B (HYG) (Table 1). Coding sequences were optimized for expression in *R. toruloides*, synthesized, and cloned into ATMT plasmid backbones described previously [22, 28], by Genscript (Piscataway, NJ).

### Transformation and screening of *R. toruloides*

Transformation of *R. toruloides* was achieved by ATMT as described previously [22]. Prior to screening, transformants were grown on yeast peptone dextrose (YPD, BD Biosciences, 242820, San Jose, CA) agar containing 300 μg/mL cefotaxime (TCI America, TCI-C2224-25G, Portland, OR) and 300 μg/mL carbenicillin (Sigma-Aldrich, C1389-5G, St. Louis, MO) to prevent the growth of *A. tumefaciens*. Plates were grown at 30 °C for three days and single colonies were inoculated into culture tubes containing 5 mL lysogeny broth (LB, Miller, VWR, J106-500G, Radnor, PA) and cultured overnight at the same temperature with shaking at 200 rpm (Adolf Kühner AG, SBM-X, Birsfelden, Switzerland). Optical density (OD) was determined by measuring absorbance at 600 nm (OD_600_) with a SpectraMax Plus 384 Microplate Reader (Molecular Devices, PLUS384, San Jose, CA). Cultures were inoculated into 10 mL YPD at a 1:100 dilution and a 20% (v/v) dodecane (Sigma-Aldrich, D221104) overlay was added to capture *ent*-kaurene. Strains were grown at 30 °C with shaking at 200 rpm for 9–10 days. After an initial round of screening, *ent*-kaurene titer of the three highest producing strains was confirmed in triplicate for each construct by growth in YPD_10_ (YPD containing 100 g/L glucose).

### Quantification of *ent*-kaurene

Following growth of engineered *R. toruloides* cultures, the dodecane phase was sampled and diluted 1:40 into dodecane containing 40 mg/L pentadecane (Sigma-Aldrich, 76510), and analyzed by gas chromatography—mass spectrometry (GC–MS) using an Agilent 6890 Plus gas chromatograph (Agilent Technologies, G1530A, Santa Clara, CA) connected to an Agilent 5973 Network mass spectrometer (Agilent Technologies, G1099A). 1 µL of each sample was injected by a CombiPal autosampler (CTC Analytics, MXY 02-00B, Zwingen, Switzerland). Analytes were separated on a DB-5MS column (30 m long, 0.25 mm internal diameter, 0.25 μm film thickness, Agilent Technologies, 122-5532) using the following oven parameters: hold for 0.5 min at an initial temperature of 100 °C, followed by a temperature ramp of 30 °C/min to 250 °C, a ramp of 10 °C/min to 270 °C, and a ramp of 30 °C/min to 300 °C. The mass spectrometer was operated in selected ion mode, with target ions (m/z) of 70, 85, 139 and 154. A standard curve was generated by running *ent*-kaurene standards at concentration range of 5–80 µg/mL. Analysis was performed using Enhanced ChemStation (Agilent Technologies, MSD Chemstation E.02.00.493) with *ent*-kaurene peak areas normalized to peak areas for pentadecane. The *ent*-kaurene standard was a gift from Dr. Joe Chappell, University of Kentucky, Lexington, KY.

### Cultivation at 2 L bioreactor scale

ABFPUB_26 was selected for growth in lignocellulosic hydrolysate at 2 L bioreactor scale. Lignocellulosic hydrolysate was prepared from corn stover by deacetylation, mechanical refining and enzymatic hydrolysis, as described previously, and is referred to as DMR-EH hydrolysate [21]. A BIOSTAT B^®^ fermentation system (Sartorius AG., Goettingen, Germany) was employed in batch mode, using a jacketed 2 L borosilicate glass vessel (UniVessel^®^, Sartorius AG, Goettingen, Germany) equipped with two 6-blade Rushton impellers, a dissolved oxygen (DO) probe (Hamilton VisiFerm DO 225, Bonaduz, Switzerland), and a pH probe (Hamilton EasyFerm Plus VP 225, Bonaduz, Switzerland). Seed cultures were grown to exponential phase and then used to inoculate 0.75 L aqueous media (75% DMR hydrolysate, 10 g/L yeast extract, and 30 mg/L cefotaxime), to which a 20% organic solvent overlay (150 mL dodecane spiked with 200 mg/L pentadecane as an internal standard) was added to capture *ent*-kaurene. The culture was grown at 30 °C with aeration maintained at 0.37 LPM and agitation at 400 rpm. After initial adjustment of the growth medium pH to 5.0, the pH was not controlled during growth. Process values were monitored and recorded using the integrated Sartorius data acquisition software (BioPAT MFCS/win). Sugar consumption, OD, and *ent*-kaurene production were measured over a period of 12 days. *Ent*-kaurene was measured by sampling of the dodecane phase, dilution, and analysis by GC–MS.

### Determination of glucose and xylose

Sugars were quantified on a Dionex Ultimate 3000 system UHPLC (Agilent Technologies) using an Aminex HPX-87H column (Bio-Rad, Hercules, CA) and Thermo Scientific™ RefractoMax 520 Refractive Index Detector (RID) held at 35 °C. Prior to analysis, samples were filtered through 0.45 μm filters (VWR Centrifugal Filters) by centrifugation at 3000×*g* for 3 min. Samples were run for 26 min using an isocratic 4 mM sulfuric acid mobile phase at 0.6 mL min^−1^ and 65 °C. Glucose, xylose, and arabinose standards were prepared and diluted to create a 7-point calibration curve ranging from 0.0625 to 4.0 mg mL^−1^. Standards were run at the beginning and end of each run, and sugar concentrations were calculated using the Chromeleon 7 software package.

### Targeted proteomics

Cultures were grown in culture tubes with 5 mL YPD_10_ medium and a dodecane overlay. Time points were collected on day 1 and 3. Protein was extracted using a method based on a previously established protocol [38]. Each cell pellet, corresponding to 6 OD units, was diluted in 200 µL of H_2_O and transferred to 2 mL pre-filled Micro-Organism Lysing Mix glass bead tubes and bead beat in a Bead Ruptor Elite bead mill homogenizer (OMNI International, Kennesaw, Georgia) at speed 5.5 for 45 s. After bead beating, the lysate was immediately placed in an ice-block and then spun into a 4 mL tube at 1,000×*g* for 10 min at 4 °C. To separate the protein, metabolites and lipids, 1 mL cold (− 20 °C) chloroform:methanol mix (prepared 2:1 (v/v)) was pipetted into a chloroform compatible 2 mL Sorenson MulTI™ SafeSeal™ microcentrifuge tubes (Sorenson bioscience, Salt Lake City, UT) inside an ice-block. The 200 µL of sample homogenate was then added to the Sorenson tube at a ratio of 1:5 sample:chloroform mix (2:1 (v/v)) and vigorously vortexed. The sample was then placed in the ice block for 5 min and then vortexed for 10 s followed by centrifugation at 10,000×*g* for 10 min at 4 °C. The upper water-soluble metabolite phase and the lower lipid soluble phase was removed. The remaining protein interlayer had 1 mL of cold 100% methanol added to each, vortexed and centrifuged again at 10,000×*g* for 10 min at 4 °C to pellet the protein. The methanol was then decanted off and the samples were placed in a fume hood to dry for ~ 10 min. 200 µL of an 8 M urea solution was added to the protein pellet and vortexed into solution. A bicinchoninic acid (BCA) assay (Thermo Scientific, Waltham, MA) was performed to determine protein concentration. Following the assay, 10 mM dithiothreitol (DTT) was added to the samples and incubated at 60 °C for 30 min with constant shaking at 800 rpm followed by the addition of 40 mM iodoacetamide (IAA) with 30 min of room temperature incubation in the dark. Samples were then diluted eightfold for preparation for digestion with 100 mM NH_4_HCO_3_, 1 mM CaCl_2_ and sequencing grade trypsin (USB, Santa Clara, CA) was added to all protein samples at a 1:50 (w/w) trypsin-to-protein ratio for 3 h at 37 °C. Digested samples were desalted using a 4-probe positive pressure Gilson GX-274 ASPEC™ system (Gilson Inc., Middleton, WI) with Discovery C18 50 mg/1 mL solid phase extraction tubes (Supelco, St. Louis, MO), using the following protocol: 3 mL of methanol was added for conditioning followed by 3 mL of 0.1% trifluoroacetic acid (TFA) in H_2_O. The samples were then loaded onto each column followed by 4 mL of 95:5 water:acetonitrile, 0.1% TFA. Samples were eluted with 1 mL 80:20 acetonitrile:water, 0.1% TFA. The samples were concentrated down to ~ 100 µL using a Speed Vac and a final BCA was performed to determine the peptide concentration and samples were diluted to 0.20 µg/µL with nanopure water for targeted proteomics analysis.

Targeted proteomics was performed via Liquid Chromatography (LC)–Selected Reaction Monitoring (SRM) approach. Five peptides per protein were initially selected based on their SRM suitability scores predicated by CONSeQuence [39, 40] software tools. All the peptides were further blasted to ensure their uniqueness to target proteins in the organism. Crude synthetic heavy isotope-labeled (e.g., 13C/15 N on C-terminal lysine and arginine) peptides were purchased from New England Peptide (Gardner, MA). Upon receiving, the crude synthetic heavy peptides were mixed together and diluted with 0.1% formic acid in 15% acetonitrile in water to obtain a nominal concentration of 3 pmol/µL for each individual peptide. The heavy peptide mixture stock solution was aliquoted and stored at − 80 °C until further use.

To develop targeted proteomics assay, all the SRM precursor-fragment ion pairs (i.e., transitions) were first analyzed using LC-SRM by spiking heavy peptides in test samples. Three transitions per peptide and three peptides per protein were selected in a final assay based on their LC performance, MS response, transition interferences, endogenous peptide detectability. Collision energies of transitions were obtained using empirical equations provided in Skyline software [41]. The selected peptides, their transitions and collision energy in the final assay are listed in Additional file 1: Table S1.

Crude heavy peptide mixture stock solution was spiked in the 0.20 µg/µL peptide samples at a nominal concentration of 37.5 fmol/µL for each peptide. LC-SRM analysis utilized a nanoACQUITY UPLC^®^ system (Waters Corporation, Milford, MA) coupled online to a TSQ Altis™ triple quadrupole mass spectrometer (Thermo Fisher Scientific). The UPLC^®^ system was equipped with an ACQUITY UPLC BEH 1.7 µm C18 column (100 µm i.d. × 10 cm) and the mobile phases were (A) 0.1% formic acid in water and (B) 0.1% formic acid in acetonitrile. 2 µL of sample (i.e., 0.4 µg of peptides) were loaded onto the column and separated using a 110-min gradient profile as follows (min:flow-rate-µL/min: %B): 0:0.4:1, 6:0.6:1, 7:0.4:1, 9:0.4:6, 40:0.4:13, 70:0.4:22, 80:0.4:40, 85:0.4:95, 91:0.5:95, 92:0.5:95, 93:0.5:50, 94:0.5:95, 95:0.6:1, 98:0.4:1. The LC column is operated with a temperature of 42 °C. The TSQ Altis™ triple quadrupole mass spectrometer was operated with ion spray voltages of 2100 ± 100 V and a capillary inlet temperature of 350 °C. Tube lens voltages were obtained from automatic tuning and calibration without further optimization. Both Q1 and Q3 were set at unit resolution of 0.7 FWHM and Q2 gas pressure was optimized at 1.5 mTorr. The transitions were scanned with a 30 min retention time window and a duty cycle of 0.8 s.

All the LC-SRM data were imported into Skyline software and the peak boundaries were manually inspected to ensure correct peak assignment and peak boundaries. Peak detection and integration were determined based on two criteria: 1. The same LC retention time and 2. Approximately the same relative peak intensity ratios across multiple transitions between the light peptides and heavy peptide standards. The total peak area ratios of endogenous light peptides and their corresponding heavy isotope-labeled internal standards were then exported from Skyline software as Ratio-to-Standard. For each peptide, the total peak area ratios of individual samples were normalized to the average total peak area ratio of all the samples. For each sample, protein abundance was calculated as an average of the normalized total peak area ratios of all three peptides of a protein.

### Measurement of transcript levels

RNA was extracted using the Maxwell 16 AS2000 instrument with a Maxwell RSC Plant RNA Kit (Promega, AS1500, Madison, WI). RNA was quantified with a NanoDrop™ 2000 (Thermo Scientific) and 25 ng was used once linear range had been identified. Relative abundance (ΔCt) of transcript levels for Gf*KS* was measured using Superscript IV One-Step RT-PCR System (Thermo Fisher Scientific, 12594100, Waltham, MA) with EvaGreen (Biotium, 31000, Hayward, CA) and a CFX96 Real-Time System C1000 Touch Thermal Cycler (Bio-Rad). Relative abundance was compared with housekeeping genes histone H3 (XP_016270870.1) and actin (XP_016271443.1). Primers are listed in Addition file 2: Table S2.

### Copy number quantification

1.5 mL aliquots were sampled from 3-day *R. toruloides* cultures, and centrifuged at 3,000×*g* for 5 min to pellet cells. Genomic DNA was harvested from the cell pellets using the Quick-DNA™ Fungal/Bacterial Miniprep Kit (Zymo Research) following the manufacturer’s instructions. Genomic DNA was quantified by using a NanoDrop™ 2000 (Thermo Scientific). To determine the relative copy number of the introduced transgenes, quantitative PCR was performed using the PowerUp™ SYBR™ Green Master Mix (Thermo Scientific) on a CFX384 Touch Real-Time PCR Detection System (BioRad) using the manufacturer’s instructions. Each reaction was set up in triplicate with 1 ng of genomic DNA as template. PCR products (approximately 1 kb) spanning the qPCR amplicons were amplified from genomic DNA for the native sequences and plasmid DNA for the transgenes. These PCR products were gel purified using the Qiaquick gel extraction kit (Qiagen) and used to generate standard curves for each qPCR primer set. Standard curves were used to calculate the copy number of transgenes relative to the native actin and GAPDH (EGU13160.1) genes. Primers are listed in Additional file 2: Table S2.

## Supplementary information

**Additional file 1: Table S1.** Peptide standards used to quantify GkKs and GgFPS(F112A) protein levels in *R* toruloides and *their fragmentation ions.*

**Additional file 2: Table S2.** Primer sequences used for q-PCR and qRT-PCR.

## Data Availability

All data generated or analyzed during this study are included in this published article.
