# Supplementary material for: Production of ent-kaurene from lignocellulosic hydrolysate in Rhodosporidium toruloides
Source: Microb Cell Fact. 2020 Feb 5;19:24. doi: 10.1186/s12934-020-1293-8 (PMC7003354; doi:10.1186/s12934-020-1293-8)
Supplement: Supplementary file 1 — Additional file 1: Table S1. Peptide standards used to quantify GkKs and GgFPS(F112A) protein levels in R toruloides and their fragmentation ions. [file 12934_2020_1293_MOESM1_ESM.pdf]

| Protein | Signature Peptide | Precursor (m/z) | Fragment (m/z) | Fragmentation Ion Type | Collision Energy |
|---|---|---|---|---|---|
| GfKS | DENGVIGFAPR | 587.8 | 930.5 | y9 | 22.3 |
| GfKS | DENGVIGFAPR | 587.8 | 660.4 | y6 | 22.3 |
| GfKS | DENGVIGFAPR | 587.8 | 547.3 | y5 | 22.3 |
| GfKS | VIDNTMGNLAR | 602.3 | 762.4 | y7 | 22.8 |
| GfKS | VIDNTMGNLAR | 602.3 | 661.3 | y6 | 22.8 |
| GfKS | VIDNTMGNLAR | 602.3 | 530.3 | y5 | 22.8 |
| GfKS | WDDEAEDYLR | 656.3 | 1125.5 | y9 | 24.6 |
| GfKS | WDDEAEDYLR | 656.3 | 766.4 | y6 | 24.6 |
| GfKS | WDDEAEDYLR | 656.3 | 695.3 | y5 | 24.6 |
| GgFPS(F112A) | NLSPVVVER | 506.8 | 785.5 | y7 | 19.5 |
| GgFPS(F112A) | NLSPVVVER | 506.8 | 698.4 | y6 | 19.5 |
| GgFPS(F112A) | NLSPVVVER | 506.8 | 349.7 | y6 | 19.5 |
| GgFPS(F112A) | DLTEDGIGHPEVGDAVAR | 617.6 | 811.9 | y16 | 19.7 |
| GgFPS(F112A) | DLTEDGIGHPEVGDAVAR | 617.6 | 761.4 | y15 | 19.7 |
| GgFPS(F112A) | DLTEDGIGHPEVGDAVAR | 617.6 | 639.3 | y13 | 19.7 |
| GgFPS(F112A) | EVLQYNAPGGK | 588.3 | 947.5 | y9 | 22.3 |
| GgFPS(F112A) | EVLQYNAPGGK | 588.3 | 834.4 | y8 | 22.3 |
| GgFPS(F112A) | EVLQYNAPGGK | 588.3 | 706.4 | y7 | 22.3 |
