# Supplementary material for: Production of ent-kaurene from lignocellulosic hydrolysate in Rhodosporidium toruloides
Source: Microb Cell Fact. 2020 Feb 5;19:24. doi: 10.1186/s12934-020-1293-8 (PMC7003354; doi:10.1186/s12934-020-1293-8)
Supplement: Supplementary file 2 — Additional file 2: Table S2. Primer sequences used for q-PCR and qRT-PCR. [file 12934_2020_1293_MOESM2_ESM.pdf]

Primer	Sequence
Actin standard curve Fw	CTGCTACGTCGCGCTCGACTTTGAG
Actin standard curve Rev	CGAAGGCGAAGTCGACATGGGCATG
Actin qPCR Fw	GATGTGGATCTCGAAGCAGG
Actin qPCR Rev	GAAGGAACAAGGGATACGTCAG
GAPDH standard curve Fw	CTTCATCAACGCGAACCAGGAGGAC
GAPDH standard curve Rev	GAGAACCGAGTACAAGCGCTCAAAGTC
GAPDH qPCR Fw	CTCGACCATCTTTGCTGTTG
GAPDH qPCR Rev	GTCTGTCCACTCTTGAAGTCG
GfKS standard curve Fw	CTTCACCAACTCGGTCCTCAACCACAAG
GfKS standard curve Rev	CCGCGCGCGATAATTTATCCTAGTTTGC
GfKS qPCR Fw	CACGCAGAAGTACCTCATCTC
GfKS qPCR Rev	AGTGGATCGAGTTGACGTTG
GgFPS standard curve Fw	TTCGTCGGCTTCTTCCCGCAGATC
GgFPS standard curve Rev	GGCGCTTGTAGATCTTCTGCGCGAG
GgFPS qPCR Fw	AGGCCATCGTCAAGTACAAG
GgFPS qPCR Rev	TCCTGGATCTGGAAGTACTCG
Actin qRT-PCR Fw	TGGAGAAGATCTGGCACCAC
Actin qRT-PCR Rev	GAGGTAGTCGGTCAGGTCAC
Histone H3 qRT-PCR Fw	ATGGCTCGTACCAAGCAAAC
Histone H3 qRT-PCR Rev	GGAGGTCCGTCTTGAAGTCC
GfKS qRT-PCR Fw	CGAAAAGTACATGCGCCTCG
GfKS qRT-PCR Rev	ATCATGTCGTAGAGCCAGCG
GgFPS qRT-PCR Fw	CTCCTGGAGTCGTCGGTCTA
GgFPS qRT-PCR Rev	GAGAAGTGCGAGAGGTCCAC
